# Automatic Detection of High-Frequency Oscillations With Neuromorphic Spiking Neural Networks

**DOI:** 10.3389/fnins.2022.861480

**Published:** 2022-06-02

**Authors:** Karla Burelo, Mohammadali Sharifshazileh, Giacomo Indiveri, Johannes Sarnthein

**Affiliations:** ^1^Klinik für Neurochirurgie, UniversitätsSpital Zürich, Universität Zürich, Zurich, Switzerland; ^2^Institute of Neuroinformatics, University of Zurich and ETH Zurich, Zurich, Switzerland; ^3^Zentrum für Neurowissenschaften Zurich, ETH und Universität Zürich, Zurich, Switzerland

**Keywords:** neuromorphic system, epileptogenic tissue, spiking neural networks, electroencephalography, epilepsy, system-on-a-chip

## Abstract

Interictal high-frequency oscillations (HFO) detected in electroencephalography recordings have been proposed as biomarkers of epileptogenesis, seizure propensity, disease severity, and treatment response. Automatic HFO detectors typically analyze the data offline using complex time-consuming algorithms, which limits their clinical application. Neuromorphic circuits offer the possibility of building compact and low-power processing systems that can analyze data on-line and in real time. In this review, we describe a fully automated detection pipeline for HFO that uses, for the first time, spiking neural networks and neuromorphic technology. We demonstrated that our HFO detection pipeline can be applied to recordings from different modalities (intracranial electroencephalography, electrocorticography, and scalp electroencephalography) and validated its operation in a custom-designed neuromorphic processor. Our HFO detection approach resulted in high accuracy and specificity in the prediction of seizure outcome in patients implanted with intracranial electroencephalography and electrocorticography, and in the prediction of epilepsy severity in patients recorded with scalp electroencephalography. Our research provides a further step toward the real-time detection of HFO using compact and low-power neuromorphic devices. The real-time detection of HFO in the operation room may improve the seizure outcome of epilepsy surgery, while the use of our neuromorphic processor for non-invasive therapy monitoring might allow for more effective medication strategies to achieve seizure control. Therefore, this work has the potential to improve the quality of life in patients with epilepsy by improving epilepsy diagnostics and treatment.

## Introduction

### Epilepsy and EEG

Epilepsy is the most common severe neurological disease. The standard initial treatment for epilepsy is anti-seizure medication (ASM), which results in seizure freedom in about 60% of patients with epilepsy (Schmidt and Schachter, 2014). For the remaining patients, in particular those with focal epilepsy, seizure freedom may be achieved after epilepsy surgery (Ryvlin and Rheims, 2016; Barba et al., 2020). The main objective of epilepsy surgery is to remove or disconnect the epileptogenic zone (EZ) (Jette et al., 2014), which is defined as the region responsible for generating seizures (Rosenow and Luders, 2001). To delineate the surgical resection, a variety of electrophysiological and imaging methods are conducted. Current methods use intracranial EEG (iEEG) and intraoperative ECoG recordings to delineate the resection area by identifying epileptiform potentials (i.e., epileptic spikes or spike-waves) (Jobst et al., 2020). These traditional biomarkers for the EZ appear in frequencies < 80 Hz. Even though epileptic spikes are sensitive and easily accessible biomarkers, they have been proven to lack a stable correlation with the disease activity (Goncharova et al., 2016; Grewal et al., 2019). Moreover, the current gold standard for assessment of any therapeutic intervention in epilepsy is self-reported seizure frequency, i.e., seizure diaries, which have also often proved to be unreliable (Cook et al., 2013; Elger and Mormann, 2013; Karoly et al., 2021). Overall, monitoring the disease state in epilepsy is the key for assessing the efficacy of ASM as well as for epilepsy surgery in achieving seizure control. However, since current methods are not reliable, more practical, and reliable biomarkers are urgently needed.

### High Frequency Oscillations as a New Biomarker for Epileptogenic Tissue

**FIGURE 1:**
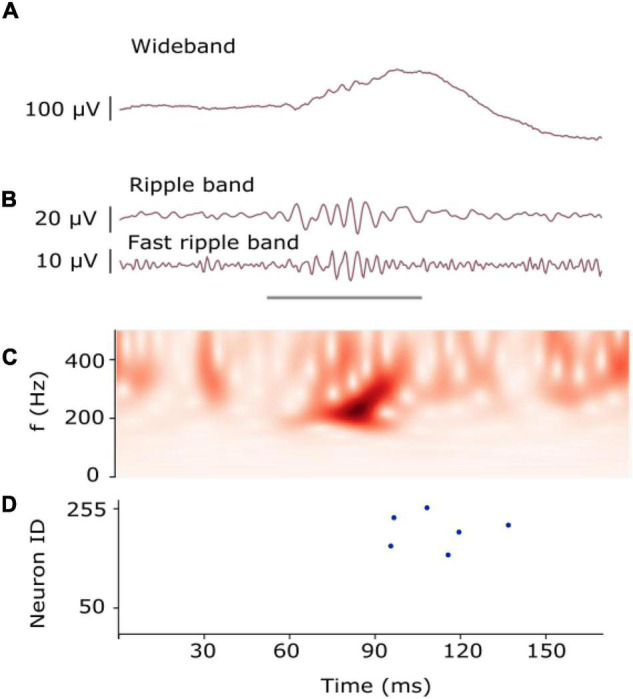
Example of an HFO. **(A)** HFO in iEEG signal as recorded. **(B)** HFO in iEEG filtered in the Ripple band (80–250 Hz) and Fast-ripple band (250–500 Hz). The gray line indicates the presence of an HFO. **(C)** The time-frequency transform of the filtered signal shows the HFO as an isolated peak. **(D)** Raster plot measured from the artificial neurons of our SNN. Multiple neurons spiked (blue dots) as they recognized the presence of the HFO [Modified from Sharifshazileh et al. (2021)].

More recently, a biomarker was proposed that appears in frequencies > 80 Hz: high-frequency Oscillations (HFO) recorded in the EEG have been proposed as a reliable biomarker for epileptogenic tissue (Jacobs et al., 2012; Frauscher et al., 2017; Jiruska et al., 2017; Jacobs and Zijlmans, 2020; Chen et al., 2021). HFO are generally described as spontaneous EEG patterns in the frequency band between 80 and 500 Hz that consist of at least four oscillations that clearly stand out of the background noise of the signal (Figure 1). Historically, HFO were first found to delineate the EZ in rat hippocampus, but their underlying physiology is not yet agreed on (Jiruska et al., 2017; Chen et al., 2021; Sarnthein et al., 2021). Nevertheless, the delineation of the EZ using HFO (Frauscher et al., 2017; Thomschewski et al., 2019) has proven to be highly predictive of seizure outcome (Fedele et al., 2017b,c, d; Boran et al., 2019b; Chen et al., 2021; Dimakopoulos et al., 2021) and therefore, may improve the outcome of epilepsy surgery. Moreover, an accumulation of recent evidence suggests that HFO are also measurable by non-invasive scalp EEG (Andrade-Valenca et al., 2011; Boran et al., 2019c; Kuhnke et al., 2019; van Klink et al., 2019; Cserpan et al., 2021a,2022a,2022b,2022c; Tamilia et al., 2021; Noorlag et al., 2022). Therefore HFO are investigated as potential biomarkers to delineate the EZ but also to monitor disease severity and treatment response (Fan et al., 2020; Jacobs and Zijlmans, 2020).

### Conventional Methods for HFO Detection

Several automatic HFO detectors have been proposed (Remakanthakurup Sindhu et al., 2020). Examples of these detectors are the Morphology detector and the Spectrum detector (Burnos et al., 2016; Fedele et al., 2017b). These detectors operate in two stages. For the first stage, both detectors find a baseline by identifying high entropy segments with low oscillatory activity when the signal is transformed in the time-frequency domain and mark the events that exceed this baseline as Event of Interest (EoI). For the time frequency analysis, the detectors use the Stockwell transform (Stockwell et al., 1996). In the second stage, the two detectors use different approaches to classify whether the EoI is an HFO or not. The Morphology detector marks the EoI as HFO using a predefined template of the morphology of an HFO (Burnos et al., 2016). The Spectrum detector assesses the time frequency signature of the EoI and marks the event as HFO if it exhibited an isolated high frequency peak (Fedele et al., 2017b). Since these detectors run in conventional computers, they require the recording data to be pre-recorded before performing the detection offline. This offline processing limits the application of HFO in clinical practice. In this project, we aim for the construction of a compact and low-power device that can be used to support the diagnostics of the disease on-line. To achieve this, we explore the capabilities of neuromorphic technology.

### HFO Detection With Neuromorphic Technology

Neuromorphic electronic circuits are brain-inspired architectures that support the implementation of spiking neuronal networks (SNNs) for solving a wide range of spatio-temporal pattern recognition problems (Brette and Gerstner, 2005; Bartolozzi and Indiveri, 2007; Indiveri et al., 2011; Chicca et al., 2014; Indiveri and Liu, 2015; Rubino et al., 2021; Bartolozzi et al., 2022). Their computation “at the edge” allows the processing of the signals being measured locally without requiring bulky computers or the need for internet connection and cloud servers. A compact embedded neuromorphic system can be designed to record EEG and detect HFO online and in real time.

Our goal was to develop spiking neural network architectures that could exploit the temporal dynamics of the silicon neurons and synapses implemented in hardware to detect clinically relevant HFO. To achieve this goal, we performed experiments with a neuromorphic hardware (Moradi et al., 2018) to determine its computational properties, its features, and its limitations. Using this information, we developed models, architectures, and full custom SNN architectures in software. For the software simulation of the SNNs, we used the Python SNN simulator Brian2 (Goodman and Brette, 2008) and the custom toolbox Teili (Milde, 2018). While accounting for the dynamics and inhomogeneous parameters of the hardware counterparts, we could demonstrate that the SNN implemented in the neuromorphic processor detected clinically relevant HFO. In a future implementation, this system might provide valuable information during surgery and simplify the collection of statistics in long-term epilepsy monitoring.

### Outline

In this review, we focus on four related publications (Table 1). In first simple approach, we used logistic regression to detect HFO in iEEG (Sharifshazileh et al., 2019). In an improved approach to detect HFO in iEEG we designed an SNN in software and validated it on hardware (Sharifshazileh et al., 2021). We next adapted the SNN to detect HFO in intraoperative ECoG (Burelo et al., 2021) and scalp EEG (Burelo et al., 2022). Different from the original publications, we compare here in detail the multiple analyses conducted in the different recording modalities (iEEG, ECoG and scalp EEG).

**TABLE 1:** SNN for HFO detection in different recording modalities.

Publication	Modality	Content	Summary of results
Sharifshazileh et al., 2019	iEEG	Measurements of hardware components HFO detection in software using logistic regression	Power consumption of each filter: 0.9 μW Static power consumption of ADM: 104 nW Power consumption of the analog headstage: 6.2 μW per channel
Sharifshazileh et al., 2021	iEEG	SNN for HFO detection in software and in hardware; core SNN; power consumption of hardware	Prediction of postsurgical seizure outcome in nine patients with 78% accuracy. Power consumption of analog headstage for pre-processing of pre-recorded iEEG: 7.3 μW per channel. Power consumption of SNN during HFO detection: 555.6 μW
Burelo et al., 2021	ECoG	Simulated SNN for HFO detection; core SNN and in-band artifact rejection	Prediction of postsurgical seizure outcome in eight patients with 100% accuracy.
Burelo et al., 2022	Scalp EEG	Simulated SNN for HFO detection; extended SNN for artifact detection	Prediction of active epilepsy in 11 pediatric patients with 80% accuracy. HFO rate correlates with seizure frequency (ρ = 0.90).

The structure of this review follows the steps needed to design a pipeline for the detection of clinical relevant HFO. In Section “Summary of main results”, we go through the pre-processing stages. In section “The signal to noise ratio (SNR) affects HFO detection”, we show how we used logistic regression to post-process the signal to find HFO and explain the reasons why we decided to build an SNN to solve this task. In section “Comparison with the clinically validated off-line automatic HFO detectors”, we go through the analysis conducted to find the architecture and parameters of an SNN that could be mapped in the neuromorphic hardware and could detect clinically relevant HFO. The Appendices contain specific details on the methods of the analyses. We briefly summarize the clinical relevance of our results in Section “Comparison of our neuromorphic SNN approach to other methods.” Finally, we discuss the challenges of HFO detection and how our approach is different from others in the literature in our vision to create a wearable medical device for epilepsy diagnostics.

## Results

### Pre-processing Stages

The signal pre-processing stages in our HFO detection pipeline are amplification, band-pass filtering, baseline detection, and transforming the continuous signal into spikes using a signal-to-spike conversion algorithm. The amplification is only in hardware and thus not further explained here. The software simulations took into consideration the characteristics and limitations of the hardware components built for each specific stage.

#### Filtering

To detect HFO, we analyzed the EEG signals in different frequency bands depending on the EEG modality. For the intraoperative ECoG data, we filtered the signal in the fast ripple band (FR) (250–500 Hz), in line with previous studies (Wu et al., 2010; Boran et al., 2019b). For the scalp EEG data, we filtered the signal in the ripple band (80–250 Hz), since it has been shown that analyzing the signal in this band gives more clinically relevant information than in the FR band (Boran et al., 2019c; Cserpan et al., 2021a). For the iEEG data, we filtered the signal in the ripple and FR bands, since the co-occurrence of HFO in these two bands showed an optimal prediction of post-surgical seizure freedom by defining an “HFO area” (Fedele et al., 2017b).

We use analog filters in hardware implementation as well as software SNN for frequency localization in HFO bands. The filters implemented in analog headstage of the neuromorphic processor DYNAP-SE2 (Sharifshazileh et al., 2021) comprise three operational amplifiers that are configured to form a Tow-Thomas resonating architecture. This configuration offers independent tuning of quality factor and center frequency of the desired frequency bands. In software simulations of the SNN, we therefore used Butterworth filters since are a good approximation of the tuned Tow-Thomas architectures implemented in hardware.

#### Signal-To-Spike Conversion

To interface and communicate with the silicon neurons in the neuromorphic processor, DYNAP-SE2 includes an asynchronous delta modulator (ADM) block at the backend of analog headstage right after the filters, which converts the analog signal into UP or DOWN digital pulses depending on the changes in the amplitude of the analog filter outputs. These pulses are from now on referred to as spikes (Corradi and Indiveri, 2015; Yang et al., 2015; Sharifshazileh et al., 2019). In the software simulations, we implemented an algorithm that emulates the behavior of the ADM circuit faithfully. In addition, to increase the efficiency and save time for tuning hyper parameters, we included a baseline detection that is used to determine the optimal spike generation thresholds automatically for this signal conversion. In Supplementary Appendix B, we explain how we detect the baseline of the signal and how the signal-to-spike conversion algorithm works both, in software and in hardware. Dynamically, the UP spikes indicate when the signal increased in amplitude more than a user-defined threshold (V_*TH,UP*_-V_*Baseline,avg*_) and the DOWN (DN) spikes indicate when the signal decreased in amplitude more than another user-defined threshold (V_*Baseline,avg*_-V_*TH,DN*_), where V_*Baseline,avg*_ is the DC component of the analog filtered signal and V_*TH,UP*_ and V_*TH,DN*_ are tunable voltages for setting spiking thresholds. One can intuitively figure out that V_*TH,UP*_ > V_*Baseline,avg*_ > V_*TH,DN*_ and we simply set thresholds for UP and DN to be equal (V_*TH,UP*_-V_*Baseline,avg*_ = V_*Baseline,avg*_-V_*TH,DN*_). With this operation principal, the occurrence rate of spikes in the ADM output depends on the derivative of the signals and user-defined thresholds. To introduce another degree of freedom for output spike rates, ADMs also feature another tunable parameter that controls the inter-spike time interval, also referred to as the refractory period, during which, the ADM cannot spike even if the signal demands spike generation.

### HFO Detection Using Logistic Regression

We first used logistic regression to study whether a simple method could achieved good performance in finding clinically relevant HFO (Sharifshazileh et al., 2019). For this analysis, we used selected data from a published data set and used it as training signal (Fedele et al., 2017a). In Supplementary Appendix A, we explain how the training signal was created. We filtered the signal in the ripple and FR band and obtained four spike trains (the UP ripple, DN ripple, UP FR, and DN FR). We first calculated the mean firing rate of each spike train separately in time windows of 45 ms (with a 10 ms overlap). The mean firing rate of the spike trains were used as “features,” the validated HFO marked in the original dataset by the Morphology detector were used as the teacher signal (Fedele et al., 2017b). In the training phase, we found the weights for each spike train to classify time windows of 45 ms as a time window containing HFO or not. For this training, we used a 20–80% cross-validation scheme. We found the weights 0.0052, 0.0086, 0.00044, and 0.0031 for the UP ripple, DN ripple, UP FR, and DN FR spike train, respectively. In the test phase we used these weights to classify time windows of 45 ms as time windows containing HFO or not in each 5-min recording of the iEEG dataset. For each patient, we calculated the HFO rate in each channel and used it to define the HFO area. In Section 3.4.1, we explain how this area is calculated. The classification task consisted of predicting the seizure outcome of the patient (seizure free or seizure recurrence) based on the overlapping of the HFO area and the resected area during surgery. The specificity achieved with this approach was 73%, which is below the one achieved by the Morphology detector in the same dataset (100%) (Fedele et al., 2017b). The poor performance of logistic regression might be because logistic regression works as an amplitude threshold and does not take into account temporal changes in the signal; these temporal changes are important when trying to find patterns in biomedical signals. To improve the performance in this classification task using the UP and DN spike trains, we decided to build an SNN and exploit its temporal properties.

### Optimal SNN Architectures and Parameters for HFO Detection

Our approach using SNNs was to explore architectures that could be mapped onto a neuromorphic processor and at the same time, could detect HFO. In designing our SNNs, we took inspiration from clinically validated automatic HFO detectors that run their analysis in conventional computers. We have specifically used the Morphology and Spectrum detectors (Burnos et al., 2016; Fedele et al., 2016, 2017b), which were used to detect HFO in the datasets used in our study. We first present the main observations of the HFO detected by these detectors, and how we used this information to define the architectures of our SNNs. In Section 3.3.1, we present the results from analyzing the UP and DN spike trains from a subset of our datasets. In the Supplementary Appendix A, we explain how we built these training signals. The results from these analyses were used to define the synaptic and neuron parameters of our SNNs. Finally, in Sections 3.3.2, 3.3.3 and 3.3.4 we present our final SNN architectures used to detect HFO in iEEG, ECoG and scalp EEG, respectively.

**FIGURE 2:**
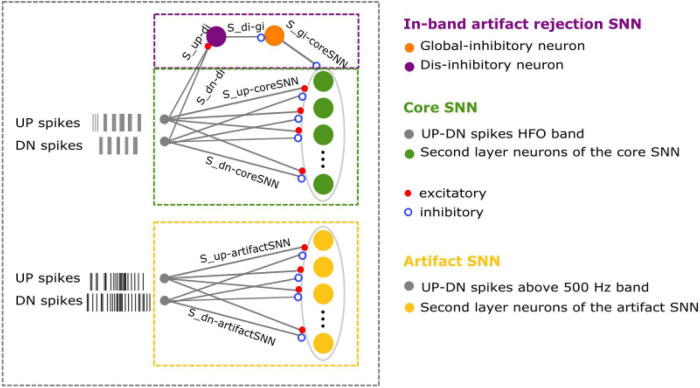
The SNN to detect HFO in iEEG, ECoG and the scalp EEG. The input to our SNNs are the UP and DN spike trains generated by converting the signals into spikes. The core SNN architecture (green box, used for iEEG) for HFO detection consists of input neurons (gray) receiving the input UP-DN spikes from the filtered signal in HFO band (ripple band and FR band for iEEG, FR band for ECoG, and ripple band for scalp EEG). These inputs project to a second layer of neurons (green) with different synaptic parameters. The core SNN can also interact with the in-band artifact rejection SNN (purple box, added for ECoG). For this interaction, the inputs of the core SNN are projected to the dis-inhibitory neuron (purple) using excitatory synapses. This neuron projects inhibitory synapses to a global-inhibitory neuron (orange), which is continuously inhibiting the second layer neurons. The role of the interneuron and the inhibitory neuron is to avoid the false detection of sharp transients. The artifact rejection SNN (yellow box, added for scalp EEG) consists of input neurons (gray) receiving the input UP-DN spikes from filtering the signal above 500 Hz. These inputs project to a second layer of neurons (yellow) with different synaptic parameters. Panels modified from Burelo et al. (2021, 2022) and Sharifshazileh et al. (2021).

For examples of HFO see the filtered signals in Figure 2. One of the characteristics in the signal that the Spectrum and Morphology detector consider to find HFO in EEG is that the signal contains four or more oscillations standing out of the baseline (Figure 1B). Since HFO present distinct frequency characteristics (Supplementary Appendix D), we investigated the frequency characteristics of our neuron and synapse models (Supplementary Appendix C). We sent spikes to our neuron model and showed that by using a specific set of time constants, the neuron was able to respond to spectral properties of single HFO (Supplementary Figure C1). To detect HFO that have a large variety of frequency characteristics, we designed an SNN with two layers. The first layer are the UP and DN spike trains and the second layer are neurons that receive the UP and DN spike trains but with slightly different synaptic parameters. The goal was to rely on the ensemble of neurons in the second layer to detect most of the clinically relevant HFO previously marked by the Morphology detector.

#### Finding the Optimal Range for the Synaptic Parameters

To find the optimal synaptic parameters for our SNNs, we followed a different approach than conventional machine learning techniques. This decision was taken since machine learning methods will result in exact values for the parameters. While in software it is possible to set exact values, in the neuromorphic hardware this is not possible due to the variability in the actual currents flowing across the transistors. This variability is called mismatch and it is inevitable (Pavasovic et al., 1994). Therefore, we instead optimized a range of values. The ranges we used were compatible with the distributions measured from the analog circuits implemented in hardware.

The synaptic parameters between the input spike trains and the second layer neurons were found heuristically by analyzing HFO and noise samples in the iEEG training signal (Supplementary Appendix A). The signal was filtered in two frequency bands (ripple and FR) and converted to spikes, which resulted in four spike trains (UP ripple, DN ripple, UP FR, and DN FR). These inputs were sent to a layer of 256 neurons. The first step to find optimal parameters was to set initial values. We calculated and analyzed the inter-spike-interval (ISI) of the spike trains. The details of this analysis are explained in Supplementary Appendix E. We observed that the interquartile range (IQR) of the ISI distribution during an HFO was 0.5–6.3 ms for the spike trains from the ripple band and 0.3–2.7 ms for the ones from the FR band. For the noise, the IQR of the ISI distribution was 0.6–10.3 ms for the spike trains from the ripple band and 0.4–4.2 ms for the ones from the FR band (Supplementary Figure E1). Therefore, for each connection between the input and a neuron in the second layer, the synaptic time constant was drawn randomly from a normal distribution with a range from 0.5 to 6.3 ms for the UP and DN spikes from the ripple signal, and from 0.3 to 2.7 ms for the UP and DN spikes from the FR signal (Supplementary Figure E2). As a second step, we analyzed the response of the neurons in the second layer to this set of parameters. The initial parameters resulted in three different type of neurons: some responded to HFO, some others responded to noise, and some others did not generate any response (Supplementary Figure E3). After we performed an automatic classification of these neurons and a cluster analysis, which is explained in detail in Supplementary Appendix E, we found that to maximize the number of neurons in the second layer that respond to HFO and remain silent otherwise, the excitatory time constant should be in a range between 3 and 6 ms. Additionally, the inhibitory time constant should be shorter that the excitatory one in a range between 0.1 to 1 ms. We called this SNN the core SNN (Figure 2).

#### The Core SNN Detects HFO in iEEG

We first used the core SNN to detect HFO in the iEEG. We filtered the signal in two frequency bands: ripple and FR (Fedele et al., 2017b). Each of these signals was converted to spikes using the ADM algorithm. The signal-to-spike threshold was set to 50% of the estimated baseline amplitude for the ripple band and 30% for the FR band. The refractory period of the signal-to-spike conversion for both bands was set to 300 μs. The ADM generated four spike trains, which were the inputs to the core SNN. The core SNN (Figure 2, green box) consists of an input layer of neurons that project the UP and DN spike trains to a second layer of neurons. For this analysis, all the preprocessing stages were performed in software. The SNN was first simulated in software and then mapped onto a neuromorphic processor to evaluate its performance in hardware (Sharifshazileh et al., 2021).

We used the SNN simulator Brian 2 and the toolbox Teili (Milde, 2018) to simulate the core SNN. In Supplementary Appendix C, we show the neuron and synapse models used in the simulations. The average time constant for all the neurons in the second layer was chosen to be 15 ms. The strength of the synaptic weights from the input layer to the second layer was set to either 1 or 2 nA. To capture the oscillations of an HFO, the polarity of these weights should be opposite. Hence, we used positive weights for the UP spikes (excitatory synapses) and negative weights for the DN spikes (inhibitory synapses). For the synaptic time constants, we first set the excitatory time constants (from the UP spikes). For each neuron in the second layer, we randomly selected a value between 3 and 6 ms. We then set the inhibitory time constant (from the DN spikes). For each neuron, we took its excitatory time constant and subtracted a randomly chosen value in the range between 0.1 and 1 ms (Sharifshazileh et al., 2021). For example, a neuron in the second layer had a time constant of 3.5 ms and its inhibitory time constant was set to 3.5–0.2 (3.3 ms). The spikes from the second layer neurons in the core SNN were used to mark HFO. Any spike within a 15 ms window indicated an HFO and consecutive windows containing spikes were concatenated to indicate the same HFO.

To validate the software simulations of the SNN in hardware, we mapped the architecture of the core SNN to the neuromorphic processor DYNAP-SE (Moradi et al., 2018), for which a working prototyping framework was available. We sent software generated UP and DN spike trains, through an FPGA *via* USB-2.0, directly to input pins of the DYNAP-SE. The FPGA is required to establish handshaking with the chip and make sure that the communication complies with address-event communication protocol.

For the second layer in the core SNN, we used a single chip-core of the DYNAP-SE, comprising 256 silicon neurons that share the same neuronal dynamics parameters. Hence, while in software we set the synaptic parameters to be random values within an optimal range (Table 2), for the hardware SNN we can only set a single value shared among all neurons, which was the mean of the optimal range optimized in software. Note that although synaptic and dynamic parameters are shared among neurons, a normal distribution of parameters (with the same mean and a different standard deviation) is maintained in hardware due to manufacturing non-idealities and mismatch among transistors and capacitors. To map the architecture, set the network parameters, and interface the input spikes, we used a high-level software-hardware interface designed in collaboration with SynSense AG., Switzerland. We then recorded the output of the silicon neurons using the same framework.

**TABLE 2:** Synapse parameters of the SNNs for HFO detection.

	Connection	Network	Name	Connection strength (fA)	Polarity	Time constant τ (ms)	Weight (fA)
1	Input UP spikes to second layer core SNN	core SNN	S_up–coreSNN_	(7–14)	exc	(3–6)	1 or 2
2	Input DN spikes to second layer core SNN	core SNN	S_up– coreSNN_	(7–14)	inh	S_up– coreSNN–_(0.1–1)	−1 or −2
3	Input UP spikes to dis-inhibitory neuron	in-band HFO rejection SNN	S_up– di_	21	exc	5	3
4	Input DN spikes to dis-inhibitory neuron	in-band HFO rejection SNN	S_up– di_	21	exc	5	3
5	Dis-inhibitory neuron to global-inhibitory neuron	in-band HFO rejection SNN	S_up–gi_	17.5	inh	20	2.5
6	Global-inhibitory neuron to second layer core SNN	in-band HFO rejection SNN	S_up– coreSNN_	24.5	inh	5	2.5
7	Input above 500 Hz UP spikes to second layer artifact SNN	artifact detection SNN	Sup-artifactSNN	(7–14)	exc	(3–6)	1 or 2
8	Input above 500Hz DN spikes to second layer artifact SNN	artifact detection SNN	Sup- artifactSNN	(7–14)	inh	Sup- artifactSNN–(0.1–1)	−1 or −2

*For HFO detection in iEEG, we used the neurons in rows 1 and 2. For HFO detection in ECoG, we added the neurons in rows 3–6.*

*For HFO detection in scalp EEG, we added the neurons in rows 7 and 8.*

*A connection between two neurons is characterized by the positive (excitatory, exc) or negative (inhibitory, inh) current in fA and the time constant.*

Any spike within a 15 ms window indicated an HFO and consecutive windows containing spikes were concatenated to indicate the same HFO. During the hardware simulations, some neurons were continuously spiking. We considered these outlier neurons to be uninformative, and therefore they were switched off for the whole study. The activity of the rest of the neurons faithfully signaled the detection of HFO.

**FIGURE 3:**
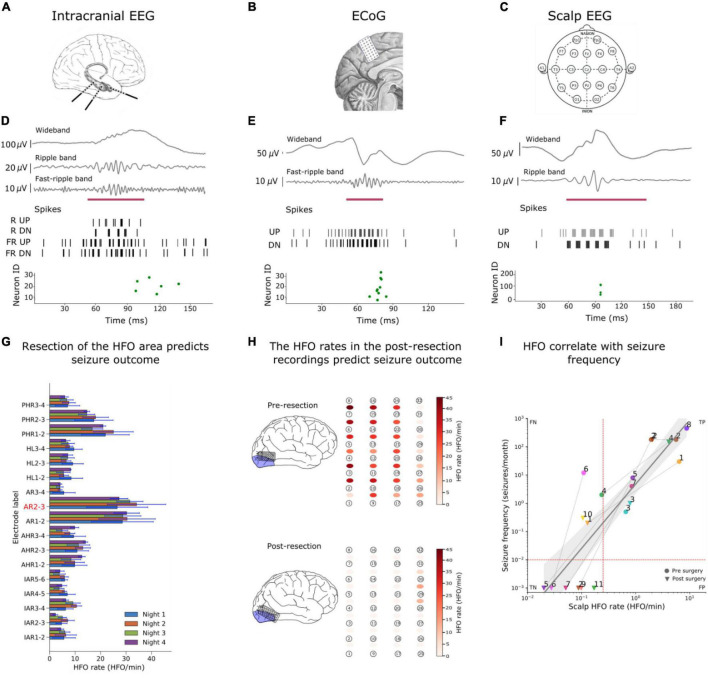
Examples of HFO detection in iEEG, ECoG and scalp EEG. **(A–C)** We detected HFO in three recording modalities. **(A)** We analyzed iEEG recordings from a patient with temporal lobe epilepsy (TLE) who was implanted with depth electrodes, **(B)** the ECoG from a patient whose surgery was guided using ECoG electrodes and (**c**) a scalp EEG from a child with drug-resistant focal lesional epilepsy. [**(D–F)** upper panels] The wideband signal was filtered in HFO bands [**(D–F)** upper panels], The filtered signals were converted into spikes [**(D–F)** middle panel] and sent as input to the SNNs. The green dots of the raster plots [**(D–F)** bottom panels] show the activity of the second layer neurons indicating the presence of an HFO in the signal, and the purple mark shows the time window that our detector marked as HFO due to this spiking activity. **(G)** We used the HFO rates found in iEEG to define the HFO area (AR2-3), which was compare to the resection area to predict the seizure outcome of the patient. **(H)** Our SNN found a high HFO rate (HFO rate > 1 HFO/min) in the pre-resection ECoG from a patient. The SNN found a HFO rate of > 1HFO/min in a patient who suffers from recurrent seizures. Hence, our SNN predicted seizure recurrence in the individual patient. **(I)** The scalp HFO rate correlated with seizure frequency of our pediatric patient and mirrored the surgical treatment response. Panels modified from Burelo et al. (2021, 2022), Sharifshazileh et al. (2021).

For both, software and hardware simulations, the core SNN successfully detected clinically relevant HFO in the iEEG dataset (Sharifshazileh et al., 2021) (Figure 3D).

#### The In-Band Artifact Rejection SNN Allows the Detection of HFO in ECoG

We next searched for HFO in the ECoG. We filtered the signal in the FR frequency band (Burelo et al., 2021). The filtered signal was converted to spikes using the ADM algorithm. The signal-to-spike threshold was set to 50% of the estimated baseline amplitude and the refractory period was set to 300 μs. The signal-to-spike conversion generated two spike trains, which we used as inputs to the core SNN. To find HFO in ECoG, we augmented the core SNN by adding an in-band artifact rejection architecture (Figure 2, purple box) (Burelo et al., 2021). This decision was taken since we observed that the signal recorded during surgery was more prone to fast transient artifacts. The in-band artifact rejection SNN consists of a global inhibitory neuron and a dis-inhibitory neuron. The global-inhibitory neuron constantly suppresses the activity of the neurons in the second layer to keep them from responding to fast transients in the signal. The UP and DN spike trains are projected to the dis-inhibitory neuron using excitatory synapses. The function of the dis-inhibitory neuron is to allow the second layer neurons to respond to HFO by suppressing the activity of the global inhibitory neuron (Burelo et al., 2021).

The parameters for the global-inhibitory and dis-inhibitory neurons were found heuristically by analyzing HFO and sharp transients in the ECoG training signal (Supplementary Appendix A). The signal was filtered in the FR band and converted to spikes, which resulted in two spike trains (UP FR and DN FR). We observed that the median duration of the HFO in the training signal was 24 ms, while the median duration of fast transients was 8 ms. Moreover, the median amount of UP-DN cycles (defined in Supplementary Appendix E) for an HFO was six cycles, while for the fast transients it was two cycles. However, a single UP-DN cycle of an HFO was shorter (2.6 ms) than a single cycle of an artifact (3.2 ms). This means that to inhibit the second layer of neurons during a fast transient, the global-inhibitory neuron must suppress their activity for at least one UP-DN cycle. The suppression of the activity of the second layer neurons during the first cycle resulted in a suppression of HFO with short duration. However, this design choice did not affect the maximum HFO rates in the benchmarking between our SNN detector and the Spectrum detector. On the other hand, to allow the second layer of neurons to respond to HFO, the dis-inhibitory neuron should start the inhibition of the global-inhibitory neuron as early as possible (i.e., after the first cycle) and should last the full duration of the HFO.

Since the dis-inhibitory neuron receives excitatory inputs from both UP and DN spikes, any activity in the signal could cause the activation of the dis-inhibitory neuron. This activation could activate the dis-inhibition of the second layer neurons and consequently, could result in an erroneous HFO detection. We avoided this dis-inhibition by using a short synaptic time constant for the connections of the dis-inhibitory neuron. Hence, the dis-inhibitory neuron was activated only during periods of elevated UP-DN spiking as it occurred during a fast transient or an HFO. Figure 2 from the Publication 3 (Burelo et al., 2021) shows an example of the interaction between the global-inhibitory, dis- inhibitory neuron and the neurons in the second layer. Adding the artifact rejection SNN to the core SNN resulted in a successful suppression of fast transients. The spikes from the second layer of the core SNN were used to mark an HFO. Any spike within a 15 ms windows indicated an HFO and consecutive windows containing spikes were concatenated to indicate the same HFO. The neurons in the second layer detected clinically relevant HFO in ECoG (Figure 3H).

#### An Extended SNN Detects HFO in Scalp EEG

We finally searched for HFO in the scalp EEG. The main difficulty in analyzing scalp EEG is that the signal to noise ratio is smaller than in iEEG and ECoG. This means that the HFO can be lost in the background noise if the parameters of the signal-to-spike conversion are not set properly. For the analysis of scalp EEG, we decided to make changes in the preprocessing stage by analyzing the impact of changing the parameters of the ADM algorithm (threshold, refractory period, and interpolation factor) on the HFO detection. For this analysis, we first filtered the scalp EEG training signal (Supplementary Appendix A) in the ripple band and converted it into spikes using the ADM algorithm. We then performed a parameter sweep over the signal-to-spike conversion threshold, refractory period, and interpolation factor. We sent the input spikes to the core SNN and compared the periods of time where the second layer neurons marked an HFO to the periods of time where the Spectrum detector mark an HFO. The optimal signal-to-spike conversion parameters were the ones that resulted in more HFO detections in accordance with the Spectrum detector and in fewer wrong detections (periods where the Spectrum detector did not mark an HFO).

To find HFO in scalp EEG, the Spectrum detector added a visual inspection of the detected HFO and performed a manual elimination (Boran et al., 2019c). This is a common step for scalp EEG since there is a larger amount of artifacts in comparison with iEEG and ECoG. In our pipeline, we also added an extra artifact rejection, however, our implementation is completely unsupervised. Our approach consists of adding an artifact detection SNN (Figure 2, yellow box) which detects artifacts in the frequency range above common HFO (> 500 Hz). The input to this network are the UP and DN spike trains encoding the scalp EEG signal filtered in the 500–900 Hz frequency band. For the signal-to-spike conversion we set the threshold to 30% of the estimated baseline amplitude and a refractory period of 300 μs. This SNN has the same architecture and parameters as the core SNN [(3–6) ms for the UP spikes and τ_*exc*_–(0.1–1) for the DN spikes], however, it receives a different input. This SNN is used in parallel to the core SNN. For the input to the core SNN and in-band artifact rejection SNN, the signal was filtered in the ripple frequency band, the signal-to-spike threshold was set to 30% of the estimated baseline amplitude, and the refractory period was set to 1 ms. The spikes from the second layer of the core SNN were used to mark an event of interest (EoI). Any spike within a 15 ms windows indicated an EoI and consecutive windows containing spikes were concatenated to indicate the same EoI. The second layer neurons in the artifact detection SNN respond to any high-amplitude signal that might have been detected as HFO by the core SNN. The 15 ms time window after a spike in the artifact detection SNN was taken as the duration of an artifact. The EoI was classified as HFO if there was no overlap between the EoI and an artifact. The goal of the artifact detection SNN is to capture oscillations that might resemble an HFO, but due to their high amplitude and frequency should be rejected instead. The artifact detection SNN successfully reduced the number of false HFO detections, which resulted in the detection of clinically relevant HFO in scalp EEG (Figure 3F).

### The SNN Detects Clinically Relevant HFO

HFO detection was performed independently for each bipolar channel in the datasets. After using the spikes from the SNN to find HFO, we calculated the HFO rate by dividing the number of HFO detected in the channel by the duration of the recording. We then tested whether the HFO rate was associated with the clinical characteristics of the patient.

#### HFO in iEEG

We analyzed the iEEG from nine patients who had been implanted with depth electrodes in the medial temporal lobe (MTL) to determine the EZ. The putative EZ was later resected in epilepsy surgery (Figure 3A). We calculated the mean HFO rate in each channel across all the recordings from a single patient. We then defined the area under the channels where the HFO rate exceeds the 95 percentile of the rate distribution as “HFO area” (i.e., channel AR2-3 in Figure 3G). Finally, we compared this HFO area with the area resected during surgery, and retrospectively “predicted” the postsurgical outcome of each patient.

The HFO area was not fully resected in a patient who suffered from recurrent seizures; we defined this correct prediction of recurrent seizures as true positive (TP = 1); The HFO area was fully resected in six patients that achieved seizure freedom; we defined this correct prediction of seizure freedom as true negative (TN = 6). The HFO area was completely resected in two patients that suffered from recurrent seizures; we defined this false prediction of seizure freedom as false negative (FN = 2). There was no case where the HFO area was not completely removed in a patient with seizure freedom (FP = 0).

Therefore, we predicted the postsurgical outcome of nine patients with TLE with a 78% accuracy and 100% specificity (Table 3). The high specificity achieved by our system indicates that the analysis of HFO using SNNs and neuromorphic technology is consistent with the current surgical planning (Sharifshazileh et al., 2021).

**TABLE 3:** Classification results of the SNN in iEEG, ECoG and scalp EEG.

Classification task and EEG modality	Seizure outcome prediction in patients implanted with iEEG. (HFO area resected →seizure freedom; HFO area not resected → seizure recurrence)			Seizure outcome prediction in patients implanted with ECoG. (max HFO rate ≥ 1 HFO/min → seizure recurrence; max HFO rate < 1 HFO/min → seizure freedom)		Active epilepsy prediction (seizures/month > 0) using scalp EEG recordings from pediatric patients (mean HFO rate > 0.25 HFO/min → active epilepsy; HFO rate < 0.25 HFO/min → seizure freedom)	
	
SNN architecture used for HFO detection	Morphology detector	Core SNN in Brian 2	DYNAP-SE hardware	Spectrum detector	Core SNN + in-band artifact rejection SNN in Brian 2	Spectrum detector	Core SNN + in-band artifact rejection SNN + artifact detection SNN in Brian 2
Specificity = TN/(TN + FP)	100 (54 100%)	100 (54 100%)	100 (54 100%)	100 (63 100%)	100 (63 100%)	67 (57 98%)	100 (69 100%)
Sensitivity = TP/(TP + FN)	0 (0 71%)	33 (1 91%)	33 (1 91%)	100 (63 100%)	100 (63 100%)	86 (57 98%)	71 (42 92%)
Negative Predictive Value = TN/(TN + FN)	67 (30 93%)	75 (35 97%)	75 (35 97%)	100 (63 100%)	100 (63 100%)	67 (22 96%)	71 (42 92%)
Positive Predictive Value = TP/(TP + FP)	–	100 (3 100%)	100 (3 100%)	100 (63 100%)	100 (63 100%)	86 (57 98%)	100 (69 100%)
Accuracy = (TP + TN)/N (%)	67 (30 93%)	78 (40 97%)	78 (40 97%)	100 (63 100%)	100 (63 100%)	85 (62 97%)	80 (56 94%)

*TP True Positive; TN True Negative; FP False Positive; FN False Negative; N = TP + TN + FP + FN = number of patients.*

*To find HFO in the iEEG dataset, we first used the SNN simulator Brian 2 and the toolbox Teili (Milde, 2018), to simulate our SNN and then we mapped the SNN in hardware using the neuromorphic processor DYNAP-SE (Moradi et al., 2018).*

*The numbers in brackets are the 95% confidence intervals (CI).*

#### HFO in ECoG

We analyzed the ECoG from eight patients whose resective epilepsy surgery was guided by intraoperative high-density ECoG. For each patient there was available a recording before (pre-) (Figure 3B) and after (post-) the resection. Since the presence of a single channel with residual HFO has been shown to predict seizure recurrence (Boran et al., 2019b), for each patient, we selected the recording channel that had the highest HFO rate in the pre- and post-resection recording. Figure 3H shows the HFO rates we found in the pre-ECoG (upper panel) and post-ECoG (bottom panel) for the same patient. The HFO rate threshold of > 1 HFO/min in unresected channels was used to “predict” seizure recurrence. Channels with an HFO rate ł 1 HFO/min in the last post-resection ECoG were defined as having residual HFO. We retrospectively “predicted” seizure freedom in patients with no residual HFO in the post-resection recording and recurrent seizures in patients with residual HFO.

The maximal HFO rate was > 1 HFO/min in all the pre-resection recordings of all eight patients [eight recordings, median duration 3.9 min, median 6.6 HFO/min, range (1.3–45) HFO/min]. In one patient we found residual HFO (HFO rate > 1 HFO/min) in the post-resection ECoG. This patient suffered from recurrent seizures; we defined this correct prediction of recurrent seizures as true positive (TP = 1) (Figure 3H). We did not find residual HFO (HFO rate < 1 HFO/min) in the post-resection ECoG of seven patients that achieved seizure freedom; we defined this correct prediction of seizure freedom as true negative (TN = 7). There was no case where we did not find residual HFO (HFO rate < 1HFO/min) in a patient with recurrent seizures (FN = 0) nor was there case where we found residual HFO (HFO rate was > 1 HFO/min) in a patient who achieve seizure freedom (FP = 0).

Therefore, we predicted the patients’ seizure outcome with a 100% accuracy and 100% specificity (Burelo et al., 2021; Table 3).

#### HFO in Scalp EEG

We analyzed the scalp EEG from 11 pediatric patients with drug-resistant focal lesional epilepsy. The presurgical scalp EEG recordings available in our dataset were part of the presurgical evaluation. While the postsurgical (Figure 3C) scalp EEG recordings were part of the follow-up after the surgery. All the scalp EEG were recorded during sleep. We then used the median HFO rate of the electrodes located on the affected hemisphere to predict whether the patient had active epilepsy (seizure frequency ≥ 1 seizure/month) or not (seizure frequency < 1 seizure/month). If the HFO rate exceeded the rate threshold, the recording was defined as a recording showing HFO. We used a rate threshold of 0.25 HFO/min which was taken from in a previous study on the same dataset (Boran et al., 2019a,c).

The scalp EEG recordings from ten patients with active epilepsy showed HFO; we defined this correct prediction of active epilepsy as true positive (TP = 10). The scalp EEG recordings from six patients who achieved seizure freedom did not show HFO; we defined this correct prediction of seizure freedom as true negative (TN = 6). The scalp EEG recordings from four patients with active epilepsy did not show HFO; we defined this false prediction of seizure freedom as false negative (FN = 4). We did not find a case where the scalp EEG recording showed HFO in a patient who achieved seizure freedom (FP = 0). Therefore, the SNN associated the HFO rate with active epilepsy with 80% accuracy and 100% specificity (Burelo et al., 2022; Table 3).

As a further result, Figure 3I shows how the HFO rate correlated with seizure frequency (ρ = 0.90, *p* < 0.0001, Spearman’s correlation). We performed a linear regression to estimate whether the HFO rate predicts the seizure frequency. The equation for the regression of seizure frequency on HFO rate becomes log_10_(seizure_frequency) = 2.27 × log_10_(HFO_rate) + 0.7 with R^2^ = 0.76. In a longitudinal approach, we observed that the change in seizure frequency (before and after surgery) was reflected in change of the HFO rate between pre- and post-operative recordings (8 cases, χ^2^_1_ = 8, *p* = 0.0047).

### Comparison of SNN With Offline Automatic HFO Detectors

In the iEEG, we compared the results obtained with our SNN to those from the Morphology detector for the individual patient and over the group of patients (Table 3). The overall prediction accuracy for our system across the nine patients is comparable to that obtained by the Morphology detector. Both detectors achieved a 100% specificity.

In the ECoG, we compared the results obtained with our SNN to those from the Spectrum detector (Table 3). The HFO rates found by the two detectors were correlated (ρ = 0.81, *p* > 0.0001 Spearman rank correlation). Moreover, the simulated SNN and the Spectrum detector reached the same “prediction” for each patient in this dataset. Even though the SNN prediction of the poor outcome (seizure recurrence) was limited to data from one patient, when not considering only post- resection but also pre-resection recordings, the SNN reached similar results as the Spectrum detector (HFOs present/HFOs not present) in all 16 recordings. Hence, the performance of the SNN on ECoG is comparable to that of the Spectrum detector.

For scalp EEG, we compared the results obtained with our SNN to those from the Spectrum detector (Table 3). The HFO rates found by the two detectors were correlated (ρ = 0.83, *p* < 0.0001 Spearman’s rank correlation). When comparing the HFO rates between affected and non-affected hemispheres, the results from the SNN did not reach statistical significance (*p* = 0.36), while in the results from the Spectrum detector there was a significant difference (*p* = 0.0003). Nevertheless, the two detectors reached agreement on the classification of epilepsy severity as they both associated the HFO rate with active epilepsy with an 80% accuracy. Moreover, both detectors established a significant correlation of HFO rate with seizure frequency (ρ = 0.90, *p* < 0.0001, Spearman’s correlation).

### Specifications of the Hardware Implementation

**FIGURE 4:**
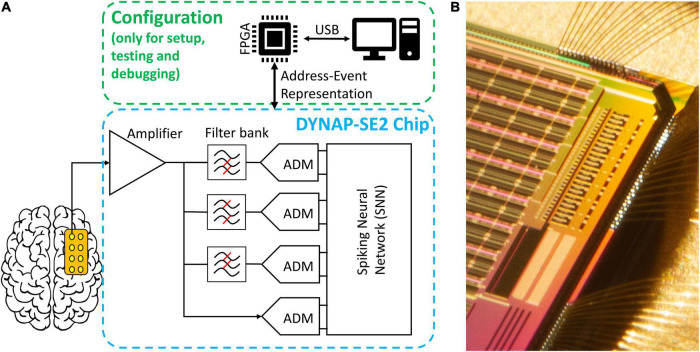
Overview of the hardware implementation of our HFO detection system. **(A)** Abstract schematic of the pre-processing and configuration pipeline. Signals from the electrodes pass through amplification, filtering and analog delta modulation (ADM) delta modulation to reach the spiking neural network (SNN). The chip can be configured, send and receive data through an FPGA daughterboard and *via* a personal computer; note that this is not required to be online during the operation of the chip. **(B)** Micrograph of the eight channels of analog headstage implemented on the top-left corner of the chip (the image is rotated 90° clockwise) and they are located right next to one of the four neural cores.

The neuromorphic hardware DYNAP-SE2 was fabricated using a standard 180 nm Complementary Metal-Oxide-Semiconductor (CMOS) process (Sharifshazileh et al., 2021). The preprocessing block that is implemented on-chip, consists of eight analog headstages each responsible for the amplification of the input signal by 20-60-dB, filtering it into three individually tunable frequency bands, and signal-to-spike conversion for individual frequency bands and raw data, with tunable spiking threshold and refractory period (Figure 4). The headstages interface with a multicore neuromorphic processor with 4 neurosynaptic cores of 256 neurons on the same chip. The processing cores are Dynamic Neuromorphic Asynchronous Processors based on the DYNAP-SE device (Moradi et al., 2018). The total chip area is 99 mm^2^. The eight headstages occupy 1.42 mm^2^ with a single headstage occupying an area of 0.15 mm^2^. For the HFO detection task, the total average power consumption of the chip at the standard 1.8 V supply voltage was 614.3 μW. The total static power consumption of a single headstage was 7.3 μW. The conversion of filtered waveforms to spikes by the ADMs consumed on average 109.17 μW. The power required by the SNN synaptic circuits to process the spike rates produced by the ADMs was 497.82 μW, while the power required by the neurons in the second layer of the SNN to produce the output spikes rates was 0.2 μW.

## Discussion

### Summary of Main Results

We designed an HFO detection pipeline using SNNs and neuromorphic technology, which is radically different from other HFO detectors. With our analysis we were able to design SNN architectures with parameters optimized for the detection of clinically relevant HFO. The HFO rates found in iEEG or ECoG predicted the patient’s seizure outcome with high specificity and accuracy in patients that underwent epilepsy surgery. Moreover, the HFO rate found by our pipeline in scalp EEG not only correlated with the seizure frequency of pediatric patients with epilepsy but it also mirrored their surgical treatment response over time.

### The Signal to Noise Ratio Affects HFO Detection

The successful detection of HFO depends on the signal acquisition quality and also on the characteristics of the automatic HFO detector. In our research, we have followed different approaches to ensure a good signal quality. The signal-to-noise ratio (SNR) and the recording setup of all the datasets used in our group are listed in Table 4. To determine the SNR, we have computed the ratio between the amplitude of an HFO found by an automatic detector (Spectrum or Morphology) and the amplitude of the signal before and after the HFO. In this study, we used the SNR information to take decisions for our SNN architectures and parameters.

**TABLE 4:** Recording setup and SNR of the iEEG, ECoG, and scalp EEG datasets.

Recording modality	Amplifier	Sampling Frequency	Signal-to-Noise Ratio (SNR)	Publication	Dataset
iEEG	Neuralynx	4 kHz, 0.5–1,000 Hz pass-band	Ripple: 3.9 FR: 2.3	Fedele et al., 2017a	Fedele et al., 2017b
	Stellate Harmonie	2 kHz, 500 Hz low pass	Ripple: 2.7 FR: 1.6	Burnos et al., 2016	
	Neuralynx	4 kHz, 500 Hz low pass	Ripple and/or FR: 3.0	Burnos et al., 2014	
ECoG	Nicolet CSeries	2 kHz, 1–800 Hz pass-band	FR: 7.3 (high-density ECoG)	Boran et al., 2019b	Boran et al., 2019a
	Nicolet CSeries	2 kHz, 1–800 Hz pass-band	FR: 2.6 (standard ECoG)		
	ISIS IONM	10 kHz, 30–2,500 Hz pass-band	FR: 0.7	Fedele et al., 2017d	
	LNA	10 kHz, 0.1–3,000 Hz pass-band	FR: 2.6		
	Nicolet CSeries	2 kHz, 1–800 Hz pass-band	Ripple: 2.8 FR: 1.1	Fedele et al., 2017c	
	ISIS IONM	10 kHz, 5–2,500 Hz pass-band	Ripple: 2.8 FR: 1.1		
	LNA	10 kHz, 0.1–3,000 Hz pass-band	Ripple: 2.7 FR: 1.6		
	Micromed	2048 kHz, 538 Hz antialiasing filter	Ripple: 10.8 FR: 8.0	Fedele et al., 2016	
Scalp EEG	LNA	10 kHz, 0.1–3,000 Hz pass-band	Ripple: 4.0	Boran et al., 2019c	Cserpan et al., 2021b
	Deltamed	10 kHz	Ripple: 3.6		

*LNA, Low Noise Amplifier.*

For the iEEG analysis, we used the dataset where the Morphology detector found HFO with an SNR of 3.9 in the ripple band and 2.3 in the FR band. The core SNN architecture used for this analysis allowed us to achieve a specificity = 100% and accuracy = 78%. Since the outcome prediction with the SNN was not inferior to that of the Morphology detector (accuracy = 67%), we conclude that our SNN was sufficient to detect enough clinically relevant HFO.

For the ECoG analysis, we used the data set obtained with the high-density electrodes as they have been shown to achieve higher SNR than standard ECoG electrodes (Boran et al., 2019b; Zweiphenning et al., 2020). In ECoG, the Spectrum detector found HFO with SNR = 7.3. The favorable SNR conditions allowed us to achieve an accuracy of 100% in the outcome prediction using the core SNN together with the in-band artifact rejection SNN.

We faced a greater challenge in the analysis of the scalp EEG, where the Spectrum detector found HFO with an SNR of 4. To find HFO in the scalp EEG, we decreased the signal-to-spike-conversion threshold. This decision allowed us to detect more HFO in the signal but it also resulted in more artifacts being falsely classified as HFO. We solved this problem by adding an automatic artifact detection stage using a separate SNN that detected transient artifacts by using the frequency band > 500 Hz, which is above the HFO band. While the Spectrum detector found a significantly higher HFO rate in the affected than in the non-affected hemisphere (*p* = 0.0003), this was not the case for our SNN detector (*p* = 0.3). This discrepancy between the detectors was more apparent in the HFO rates of patients with a deep-seated lesion, i.e., where the recording EEG channels were located far from the HFO generator. In these patients, both detectors found smaller HFO rates than in patients with a more superficial lesion, suggesting a lower SNR in these recordings. This observation suggests that our SNN may be more prone to low SNR than the Spectrum detector. Nevertheless, our SNN detector was able to associate the HFO rate with active epilepsy with an 80% accuracy.

Overall, across all the recording modalities (iEEG, ECoG, and scalp EEG), we demonstrated that the signal quality and our SNN architecture and parameter decisions were sufficient to characterize the clinical state of patients with epilepsy (i.e., determine epilepsy severity, seizure frequency, or predict the seizure outcome of a patient that underwent surgery) using neuromorphic technology.

### Comparison With the Clinically Validated Off-Line Automatic HFO Detectors

All the datasets used in this work were previously analyzed for HFO by automatic detectors that obtained clinically relevant results (Morphology and Spectrum) (Burnos et al., 2016; Fedele et al., 2016, 2017c). Since for all the analyses we used pre-recorded data, we were constrained by the signal acquisition quality of the recordings. For the analysis of this retrospectively collected data, offline automatic detectors like the Spectrum and Morphology detectors have the advantage of *post hoc* signal processing analyses. For example, both detectors perform a time-frequency transformation across the whole signal only to define a baseline for detecting HFO. This analysis consumes a much higher power with conventional computers (order of Watt) and with off-the-shelf analog components and custom processors (order of mW) compared to the power consumption of our neuromorphic processor (order of μW). In designing our SNNs, we could not apply the same techniques as the offline automatic detectors to facilitate the HFO detection. However, we investigated the frequency characteristics of our neurons and synapses and chose the parameter range that resulted in the detection of HFO with a variety of frequency characteristics. Note that we did not aim to have a one to one agreement with these well-established automatic detectors. Rather, our SNNs were built with the aim to analyze the data online with our neuromorphic device. Overall, there was a comparable level of prediction accuracy between the previously validated off-line automatic detectors and our SNNs (Table 3).

We investigated the frequency characteristics of our neuron and synapse models (Supplementary Appendix C). We sent spikes to our neuron model and showed that by using a specific set of time constants, the neuron was able to respond to spectral properties of single HFO (Supplementary Figure C1). To detect HFO that have a large variety of frequency characteristics, we designed an SNN with two layers. The first layer are the UP and DN spike trains and the second layer are neurons that receive the UP and DN spike trains but with slightly different synaptic parameters. The goal was to rely on the ensemble of neurons in the second layer to detect most of the clinically relevant HFO previously marked by the Morphology detector.

### Comparison of Our Neuromorphic SNN Approach to Other Methods

In this project, we have designed an embedded system for processing EEG to demonstrate the clinical relevance of HFO and to aid in the treatment of patients with epilepsy. Although other embedded systems and VLSI devices have been designed in the past for the processing of EEG signals (Yoo et al., 2012; Van Helleputte et al., 2014; Feng et al., 2018; Burrello et al., 2019), their power consumption is in the order of mW compared to the order of μW consumption of our whole system (headstage + processor). Moreover, they usually lack the co-integration of an analog headstage with the processor and use off-the-shelf analog components as cost-/time-saving measures since perfecting the design of these blocks requires excessive amount of time, financial resources and highly specialized labor force. Separating the signal encoding stage from the processing stages allows the implementation of sophisticated signal processing techniques and machine learning algorithms (Feng et al., 2018; Burrello et al., 2019; Zanghieri et al., 2021). However, using off-the-shelf platforms for signal encoding or processing leads to much higher power consumption and bulky platforms that make the design of compact and portable embedded systems more challenging.

To overcome these issues, we have focused on designing a device using neuromorphic technology. Neuromorphic processors offer the possibility to develop low-power and compact devices, which results from their capability to carry out computation “at the edge” (Pei et al., 2019; Yang et al., 2020, 2021; Rubino et al., 2021; Sharifshazileh et al., 2021; Bartolozzi et al., 2022). While the field of neuromorphic is mainly focused on the design of multiprocessors, in this study we have augmented the capabilities of neuromorphic technology by designing an analog headstage for the pre-processing of biomedical signals that are inherently desirable for mixed-signal neuromorphic processing due to their sparse activity and low-frequency components. This headstage was carefully designed to be compatible with the neuromorphic processor and therefore our project results in a system that preserves the compactness and power consumption advantages that neuromorphic approaches offer.

As a consequence of our decision to use neuromorphic hardware, our signal processing pipeline to find HFO is also different from other HFO detectors proposed in the literature (Fedele et al., 2016, 2017b,2017c; Weiss et al., 2018; Boran et al., 2019b; Nariai et al., 2019; Remakanthakurup Sindhu et al., 2020). The main difference is the use of neuromorphic SNNs. These architectures emulate many of the features found in biological neural processing systems, such as the temporal dynamics of the neurons and synapses, or the variability in their time constants, refractory periods, and synaptic weights. Therefore, instead of using conventional machine learning methods, where the convention is to use neurons with homogeneous parameters and a learning algorithm to determine the weights of static synapses, we decided to exploit the temporal dynamics and variability of the synapse and neuron elements. Another noticeable difference is the preprocessing and data conversion scheme in which instead of synchronous digitization, asynchronous delta modulation is employed for the benefit of interfacing with the SNN directly on-chip. Furthermore, since conventional binary digitization is not performed, we are using second-order analog filters in real-time and not complex high-order digital filters are usually implemented offline in software. Overall, our approach was to tune the parameters governing the dynamics of the synapses and exploit their variability to create an ensemble of weak classifiers that can reliably and robustly detect HFO. Although it is possible to use more complicated neural networks using multiple layers and backpropagation to learn more complicated network parameters, these approaches usually require a great amount of data to train their models. Even though we have analyzed several hours of EEG recordings to find HFO, we did not use a predefined set of HFO (i.e., HFO marked by other detectors or by visual inspectors) as ground truth. In our first study, we used HFO markings from the Morphology detector only as a guideline, and compared our results against the clinical outcome of the individual patient. Therefore, our sample number (number of patients) is probably too low to use deep learning techniques. In contrast, for our approach the small number of patients did not represent a problem. In fact, we were able to use the properties of the silicon circuits in our favor. For example, instead of carefully determining values for the synaptic and neuron parameters, we sampled those values from distributions with hardware-compatible ranges and optimal mean values. We showed that the mismatch among the silicon neurons in the hardware resulted in a key feature to generate the normal distribution of parameters without manually defining the parameters found in software without requiring extra memory to allocate these values. Therefore, we have developed an HFO detection pipeline that achieves a high accuracy in predicting the clinical outcome of patients with epilepsy without the need of power-hungry methods running in cloud servers or GPUs. Additionally, this approach of setting a range of parameters allows the network to be robust against signal noise, which is a typical characteristic of biomedical signals.

### Implementation in Our Neuromorphic Hardware

All our analyses were motivated by future implementations of an HFO detector in neuromorphic hardware. All the pre-processing stages (amplification, filtering, and signal-to-spike conversion) were tested individually using a pre-recorded iEEG signal. To assess the compatibility of the simulated SNN with the neuromorphic processor, we performed HFO detection in iEEG using the neuromorphic processor DYNAP-SE and the core SNN architecture. Both SNNs performed equally well and lead to the same clinically relevant results. For the HFO detection in ECoG and scalp EEG, we only simulated the SNNs. For the parameters and architectures of the in-band artifact rejection and artifact detection SNN, we followed a similar approach as with the simulated core SNN and carefully defined the range of parameters. Therefore, also these SNNs can easily be implemented in hardware with only slight modifications. We hypothesize that the same clinically relevant results would be achieved with these SNNs in hardware.

Performing HFO detection with neuromorphic technology results in a low-power solution because these circuits perform spike-based processing without a fixed sampling rate. The bulk of consumed power throughout the pipeline is thus of the dynamic natures cause by charging and discharging capacitors (*P* = C_*tot*_.V_*dd*_^2^). This notion is maintained in the signal acquisition blocks. While amplifiers and filters run continuously (and consume very little static power), data conversion using ADMs is very low-power since HFO are sparse events and generating ADM spikes is highly input-dependent. And for the HFO detection, the circuits in the SNN only consume power when events arrive from the ADM. In our first two studies (Sharifshazileh et al., 2019, 2021), we have demonstrated that the components of the analog headstage in DYNAP-SE2 can perform signal amplification, band-pass filtering, and signal-to-spike conversion on eight input channels simultaneously with a power consumption of 58.4 μW. The power required for the SNN to process the input spikes and generate output spikes during the HFO detection task was 555.6 μW.

DYNAP-SE2 was built with the idea to have for the first time a preprocessing signal acquisition block and a neuromorphic processor on the same silicon die for the processing of biomedical signals. For an HFO detection device, a new generation of this device should be designed with slight adaptations. The software simulations for the analysis of this review therefore provide guidelines and specifications for the design of these future neuromorphic processors.

A clear improvement will be adding the baseline detection step to the next generation of hardware. This stage was very important in our HFO detection software pipeline since it controls and optimizes the number of input spikes going into the SNNs for best HFO detection performance. However, in the current hardware, the ADM does not calculate the baseline to set the threshold automatically and these parameters need to be manually tuned for individual ADMs by the user, resulting in a time consuming trial and error phase before starting the analysis. This baseline detection step was carefully designed and implemented in simulations to be compatible for future implementation in hardware.

Moreover, the simulations of the SNN can serve as a guideline of the number of neurons that are required to solve the HFO detection task. This information might help in reducing even further the size and power consumption of the device.

We envision a real-time HFO analysis “at the edge” that in addition to the pre-processing stages and the HFO detection with the SNN, it also features a wireless transmission of a flag to a storage device at the time of HFO occurrence. Using a miniature Bluetooth low-energy transmitter, we can have a device that operates continuously for 12 days using a battery with a capacity of 660 mAh and weighting only 1.8 g.

### Outlook on Clinical Value of HFO

The analysis of HFO in EEG faces constraints of spatial coverage and sampling imposed by the placement and size of the electrodes used for the recordings. We have overcome these limitations by following similar approaches as the ones used in previous studies (i.e., using recordings from high-density electrodes and low-noise amplifiers, and performing test-retest analysis of our results). We have compared our HFO rates against seizure outcome. For the clinical use of HFO, a prospective definition of a clinically relevant HFO should be tested in a larger patient cohort from multiple centers (Dimakopoulos et al., 2021, 2022). This HFO analysis should be fast, reliable, and able to perform an unsupervised automatic HFO detection. We have shown that our compact and low-noise neuromorphic processor has the potential to analyze EEG signals, recorded from different clinical settings, on-line and in real-time. Therefore, it can be used for a standardized multi-center HFO analysis, thereby increasing the value of HFO in the diagnostics and therapy monitoring of focal epilepsy.

### Impact of SNN for Epilepsy Diagnostics

We have demonstrated that neuromorphic SNN architectures and hardware-compatible parameters used for analyzing iEEG, ECoG and scalp EEG are able to find clinically relevant HFO. Our HFO detection pipeline can be fully implemented in our device DYNAP-SE2, which demonstrates that common pre-processing stages like low-noise amplification, filtering and signal transformation using ADMs can be implemented in the same silicon die alongside a multi-core neuromorphic processor. This computation “at the edge” allows the on-line and real-time processing of locally measured biomedical signals. Since the device is compact, battery powered, and does not interfere with other electronic equipment, it can be used for long-term recordings for monitoring and assisting epilepsy treatments. For example, our device can provide real-time feedback about the regions with high HFO activity during surgery using ECoG recordings. This feedback might guide the surgeon in ambiguous cases, improving the success rate of epilepsy surgery. Even more relevant is the application of our device in scalp EEG, since this modality is accessible to a broader population. We envision a device that can be used to monitor the disease state and more generally to simplify the collection of statistics in long-term epilepsy monitoring, thereby supporting the clinicians in the diagnostics of this unpredictable disease (Hubbard et al., 2021).

## Conclusion

We developed a fully automated HFO detection pipeline using for the first time SNN and neuromorphic intelligence. We demonstrated that our pipeline can be applied to EEG recordings from different modalities and showed that our analysis leads to clinically relevant HFO. While we focused on the software simulations, we have also demonstrated that our pipeline can be implemented in a neuromorphic device, which can perform the pre- and post- processing stages on the same chip for the analysis of bio-signals with very low power and in real time. The software simulations not only allowed us to find the best SNN architectures for HFO detection but they also provided solutions for setting the hyperparameters of the analog circuits implemented in hardware. The results of our automatic HFO detection together with the design of our neuromorphic device ensures a prospective, standardized, and bias-free definition of clinically relevant HFO. The analysis of ECoG and iEEG with our device provides a further step toward a real-time detection of HFO in the operation room, which may improve the seizure outcome of epilepsy surgery. Moreover, given the widespread access to non-invasive EEG, our approach also provides a further step toward non-invasive therapy monitoring, which might benefit a broader population of patients affected by epilepsy.

## Author Contributions

KB performed simulations and prepared figures. MS designed hardware and prepared figures. GI and JS supervised the analyses. All authors wrote and reviewed the manuscript.

## Conflict of Interest

The authors declare that the research was conducted in the absence of any commercial or financial relationships that could be construed as a potential conflict of interest.

## Publisher’s Note

All claims expressed in this article are solely those of the authors and do not necessarily represent those of their affiliated organizations, or those of the publisher, the editors and the reviewers. Any product that may be evaluated in this article, or claim that may be made by its manufacturer, is not guaranteed or endorsed by the publisher.

## Abbreviations

ADM: Asynchronous delta modulator
ASM: Anti-seizure medication
ECoG: Electrocorticography
EEG: Electroencephalography
EoI: Event of interest
EZ: Epileptogenic zone
FN: False negative
FP: False positive
FR: Fast ripple
HFO: High frequency oscillations
iEEG: Intracranial EEG
ILAE: International League Against Epilepsy scale
IQR: Interquartile range
ISI: Inter-spike-interval
NPV: Negative predictive value
PPV: Positive predictive value
SNN: Spiking Neural Network
TLE: Temporal lobe epilepsy
TN: True negative
TP: True positive.
