# Supplementary material for: Automatic Detection of High-Frequency Oscillations With Neuromorphic Spiking Neural Networks
Source: Front Neurosci. 2022 Jun 2;16:861480. doi: 10.3389/fnins.2022.861480 (PMC9205405; doi:10.3389/fnins.2022.861480)
Supplement: Supplementary file 1 [file Data_Sheet_1.docx]

## Appendix A. Data and training signals

**Patients and EEG recordings**

For all our analysis, we included recordings from patients with drug-resistant epilepsy who underwent epilepsy surgery. To find HFO in iEEG, we analyzed the recordings during deep sleep from 9 patients with temporal lobe epilepsy (TLE) who were implanted with deep electrodes (total data duration 18 hours, mean duration of a single recording 5 min) (Fedele, T.S. Burnos, 2017). To find HFO in ECoG, we analyzed the pre- and post-resection recordings from 8 patients whose surgery was guided using high-density ECoG (total data duration 73 min, mean duration of a single recording 4.5 min) (Boran, E.J. Sarnthein, 2019). To find HFO in scalp EEG, we analyzed recordings during deep sleep from 11 children and adolescents with drug-resistant focal lesional epilepsy (total data duration 544 min, mean duration of a single recording 5 min) (Boran, E.J. Sarnthein, 2019). All patients had a follow-up period after surgery ≥ 11 months (median follow-up 32 months) (Petrik et al., 2021

).

**Training signals for HFO detection with SNNs**

To find optimal parameters for our SNN architectures, we constructed training signals that included examples of noise and examples of HFO detected by a clinically validated HFO detector (Burnos, 2016; Fedele, T.S. Burnos, 2017)).

To calibrate the parameters of the core SNN, we used a small subset of the iEEG dataset with 102 HFO markings by the Morphology detector (Fedele, T.S. Burnos, 2017). We chose three recording intervals where the Morphology detector found a high HFO rate. These recordings belonged to the same patient. From these signals, we chose the snippets where HFO were marked ± 25 ms. We filtered the signal of each snippet in the ripple and FR band and concatenated them to form an iEEG training signal, which had a duration of 33 seconds.

To calibrate the parameters of the in-band artifact rejection SNN, we used a small subset of the ECoG dataset containing 11 snippets of clinically relevant HFO marked by the Spectrum detector and 11 snippets of signal containing sharp transients (Boran, E, 2019). All the snippets were of 50 ms wide. We filtered the signal of each snippet in the FR band and concatenated them to form the ECoG training signal, which had a duration of 4.4 seconds.

To calibrate the parameters for detecting HFO in scalp EEG, we used a 5-min interval of a single recorded channel from a single patient as the scalp EEG training signal (Boran, E.J. Sarnthein, 2019).

## Appendix B. Signal-to-spike conversion

Contradictory to hardware ADM implementation, in software simulations we first up-sampled the signal to an arbitrary value of 35 kHz. The upsampling frequency should be high enough(10 times larger than the bandwidth of the raw input data is a good starting point) to avoid a constraint on the time stamps where a spike can occur since the hardware ADM works without a clock and therefore, it can generate a spike at any point in continuous time (Sharifshazileh, 2019; Sharifshazileh, 2021). The signal-to-spike conversion algorithm gets the value of the upsampled input signal at each time point and compares the amount of change since last time a spike was generated, to the UP (or DOWN) threshold. If the positive or negative amplitude change is larger than these thresholds, the algorithm stores the current timestamp in an UP (DN) vector. After a spike is marked, the algorithm takes the current value of the signal as a base for the next threshold comparison. Another important step in this algorithm is to model the time that silicon neurons need before they can generate a new spike. Both in hardware and software, we call this time period ‘refractory period’. In software, once the algorithm marks a spike we simply skip a set number of timestamps that correspond to the refractory period before performing the threshold comparison again. At the end of the signal-to-spike conversion, we export two vectors, the UP and DN spike trains.


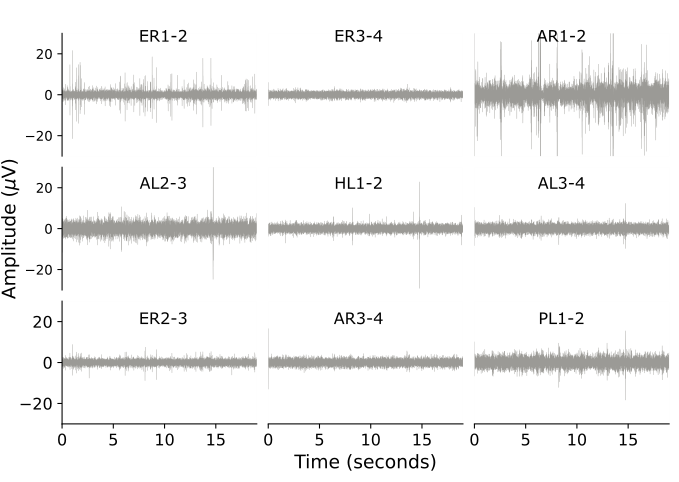


**Figure B1.** Variation of the noise floor in signals within the same recording. First 20 seconds from the iEEG signal of 9 recording channels. The signals are from the same recording interval and patient. The noise floor of the signal varies across recordings. Note that using a fixed threshold to convert these 9 signals into spikes, would results in more spikes for channel AR1-2 than for channel ER3-4.

**Baseline detection**

Biological data is sensitive to noise, which can be generated by electronic components in the recording headstage, the electrical gird lines, wireless and high-power electrical equipment, patient movement, or placement of the electrodes. This noise impacts the analysis of biomedical signals. **Figure B1** shows how the noise floor varies in the recordings of 9 electrodes from the same interval recording and patient. In the HFO detection pipeline, we aim to detect oscillations that stand out of the baseline. Hence, it is important to estimate the noise floor amplitude before converting the continuous signal into spikes. For this estimation, we selected a 1 s time window, stored the maximum signal amplitudes of consecutive non-overlapping time sub-windows of 50 ms, and took the mean of the lowest quartile as the baseline amplitude.

**Signal reconstruction**

When we convert the signal into spikes, some information of the original signal will be lost. For example, during periods bellow the signal-to-spike threshold. In the previous step, we estimated the baseline, which contains the information of the noise floor amplitude. To detect HFO, we do not need the signal information during the baseline periods, however, using the average baseline amplitude value directly as signal-to-spike threshold resulted in signal loss during HFO detection experiments (**Fig. B2d**). In this step we found a fraction of this baseline that resulted in the detection of HFO. This would ensure correlation between average baseline amplitude and the delta modulation thresholds while maintaining the integrity of HFO for detection. We refer to this fraction as ‘scaling factor’. We set the signal-to-spike threshold using different scaling factors of the baseline, converted the signal into spikes, and used the generated spikes to reconstruct the signal. We then qualitatively compared the reconstructed signal with the original one and chose the scaling factor that recovered the relevant signal information during the HFO minimizing redundant information from the noise floor. For the signal filtered in the ripple band a scaling factor of 0.5 resulted in a good reconstruction of the HFO oscillations while a smaller scaling factor generated more spikes without adding more information. For the signal filtered in the FR band a scaling factor of 0.5 was not enough to reconstruct the HFO oscillations. We found better results using a scaling factor of 0.3.


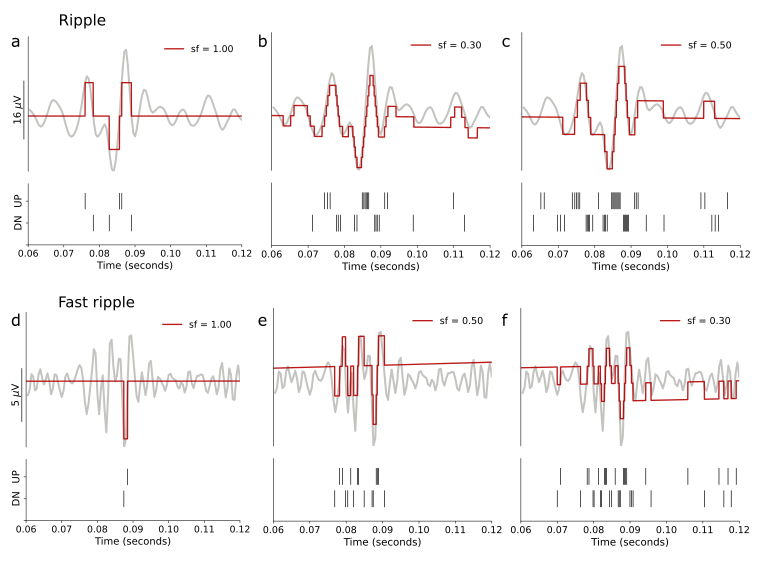


**Figure B2.** Comparison of the original iEEG signal with the reconstructed signal using spikes. In grey, the iEEG signal filtered in the ripple band (a-c top) and in the FR band (d-f top) during an HFO with their respective reconstructed signal (red). The reconstructed signal was recovered from the spikes (a-f bottom) obtained from the signal-to-spike conversion algorithm.

## Appendix C. Simulation of SNNs in software

The neuromorphic processor contains silicon neurons and synapses that emulate the biophysical properties of biological neurons (Bartolozzi, 2007; Chicca, 2014; Indiveri, 2011). For the software simulation of the SNNs, we used the Python SNN simulator Brian2 (Goodman, 2008) and the custom toolbox Teili(Milde, 2018). This toolbox contains the equations that describe the neuromorphic circuits of the silicon neuron and synapses. The model of the silicon neurons reproduce the properties of Adaptive-Exponential Integrate and Fire (AdExp-I&F) neuron models (Brette, 2005). The equations that describe the behavior of AdExp-I&F neurons are the following:

$$\tau_{mem}\frac{d}{dt}V_{mem}\left( t \right)=-V_{mem}\left( t \right)+I_{syn}\left( t \right)- v_{ahp}\left( t \right)+f\left( V_{mem} \right) (Eq. C1)$$

$$\tau_{ahp}\frac{d}{dt}v_{ahp}\left( t \right)=-v_{ahp}\left( t \right)+w_{ahp}\partial_{spk}\left( t \right) (Eq. C2)$$

Where $V_{mem}$ represents the neuron’s membrane potential, $f\left( . \right)$is an exponential function of $V_{mem}$ with a positive exponent (Brette, 2005), $v_{ahp}$ represents the after-hypolarizing term that is increased with every output spike, and which has a negative feedback onto the membrane potential, typical of spike-frequency adaptation mechanisms (Ha and Cheong, 2017). The term $\partial_{spk}\left( t \right)$ is 1 when the neuron spikes and zero otherwise. The terms $\tau_{mem}$ and $\tau_{ahp}$ represent the time constants of the membrane potential and after-hypolarizing potential respectively. The term $I_{syn}\left( t \right)$ represents the total weighted sum of the synaptic inputs, which can be either excitatory or inhibitory ($I_{syn}\left( t \right)= I_{exc}\left( t \right)- I_{inh}\left( t \right)$).

The model for the silicon synapse exhibits first order temporal dynamics (Bartolozzi, 2007). The equations that govern the dynamics of the synaptic excitatory and inhibitory circuits are, to first order approximations:

$$\tau_{exc}\frac{d}{dt}I_{exc}\left( t \right)+ I_{exc}=w_{exc}\partial_{UP}\left( t \right) (Eq. C3)$$

$$\tau_{inh}\frac{d}{dt}I_{inh}\left( t \right)+ I_{inh}=w_{inh}\partial_{DN}\left( t \right) (Eq. C4)$$

Where $\tau_{exc}$ and $\tau_{inh}$ represent the time constants of the synapses, $w_{exc}$ and $w_{inh}$ their weights. The terms $\partial_{UP}\left( t \right)$ and $\partial_{DN}\left( t \right)$ are one during an UP and DN input spike respectively, and zero otherwise

The values used for these parameters are listed in **Table 2**. The software simulations take into account the circuit properties which are based on a ‘current-mode’ design, therefore, the parameters used in the SNNs are given as currents.

**Figure C1a** shows the behavioral simulation results for the normalized steady-state response of the synapse model to spike trains encoding an input sine wave, as a function of sine wave frequency. As expected, the synapse model reproduces a standard low-pass filter behavior, also for spiking inputs. By combining the response of an excitatory synapse with an inhibitory synapse and appropriately choosing their time constants, we can effectively design neurons that are tuned to the spectral properties of the signal (**Figure C1b**).


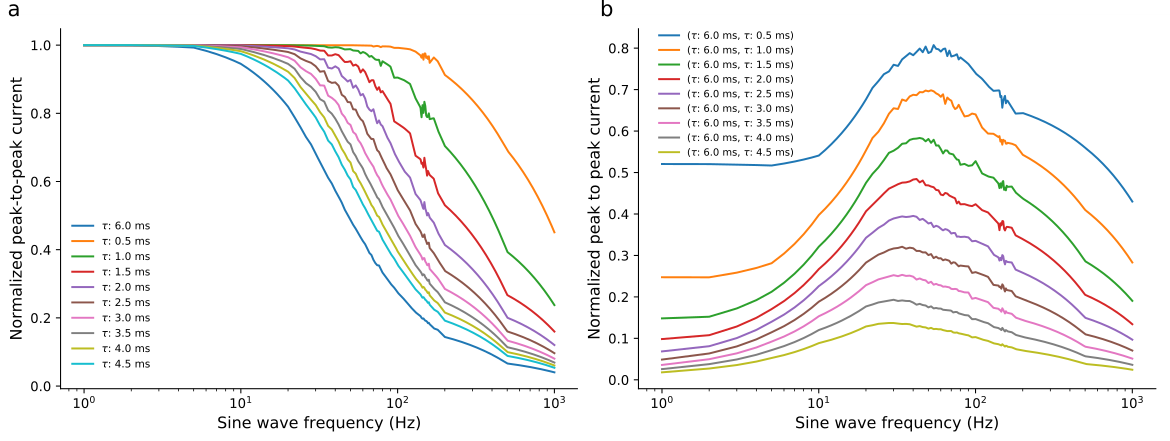


**Figure C1**. Behavioral simulation results for the normalized steady-state response of the silicon synapses to spike trains encoding an input sinewave, as a function of sinewave frequency (a). The silicon synapse is able to reproduce a standard low-pass filter behavior for spiking inputs. (b) A first-order band-pass filter results from subtracting the time responses to sine waves of varying frequencies of a single excitatory synapse with a given time constant with the time response of an inhibitory synapse with a different time constant (Figure taken from(Sharifshazileh, 2021)).

## Appendix D. Architectures for the SNN

**Exploring SNN architectures to detect clinically relevant HFO**

For our first approach to detect HFO with SNNs, we designed a single perceptron neuron that received as input the UP and DN spike trains. To capture the oscillations of an HFO, we used excitatory synapses for the connections between the UP spikes and the perceptron neuron, and inhibitory synapses for the connections coming from the DN spikes.


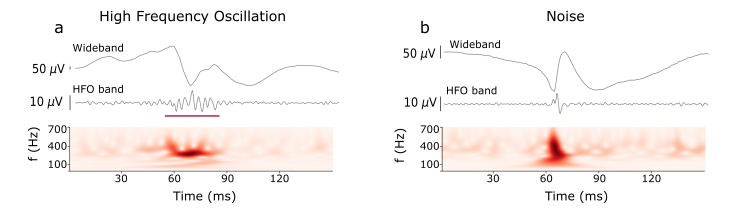


**Figure D1**. Frequency response of EEG during HFO and noise. We filtered two snippets of the training signal in the FR band and used the Stockwell transform to analyze their time-frequency characteristics. The frequency characteristics of an HFO (a) shows a clear and localized peak in the frequency 300 Hz. This clear peak is not visible in the frequency characteristics of the noise example (b), rather it shows that all the frequencies in the FR band are equally present in the pattern.

When we analyzed the marked HFO in the iEEG training signal, we observed that the frequency footprint of these HFO was well determined. For example, **Figure D1** shows the frequency response of the EEG signal filtered in the FR band during an HFO (**Figure D1a**) and during noise (**Figure D1b**). For the HFO, we observed a peak frequency of 300 Hz, while there was not a clear peak frequency in the frequency response of the noise sample. To detect the HFO in **Figure D1a**, the synaptic time constants of these connections should allow the neuron to elicit a respond to the signal oscillating at around 300 Hz and remain silent for other frequencies. Note that a single neuron tuned to a specific frequency will not be able to detect HFO oscillating at a different frequency (**Figure C1)**. To find HFO with different peak frequencies, we need many neurons tuned to different frequencies. To achieve this, we sent the UP and DN spikes to a layer of perceptron neurons (second layer of neurons). Each neuron received the same input but with different synaptic parameters.

## Appendix E. Parameters for the SNNs

**Analysis of the ISI in the UP and DN spike trains**

We filtered the iEEG training signal in the ripple and FR band and converted them to UP and DN spikes. We then analyzed the differences between the signal containing an HFO and the signal containing noise by comparing the temporal characteristics (i.e. ISI) of the UP and DN spike trains obtained when the signal was converted into spikes.

**Figure E1**. Shows the distribution of ISI of UP and DN spikes for all the HFO and noise in the training signal. The ISI distribution of UP spikes does not differ much from the ISI distribution of the DN spikes for the HFO nor for the noise. For the HFO in the ripple band, the median ISI for the UP spikes was 1.09 ms (IQR 0.51-6.34 ms) and for the DN spikes was 1.43 ms (IQR 0.57-6.74 ms). For the noise in the ripple band, the median ISI for the UP spikes was 3.46 ms (IQR 0.6-10.26 ms) and for the DN spikes was 4.4 ms (IQR 0.7-10.26 ms). For the HFO in the FR band, the median ISI for the UP spikes was 0.57 ms (IQR 0.29-2.69 ms) and for the DN spikes was 0.63 ms (IQR 0.29-2.69 ms). For the noise in the FR band, the median ISI for the UP spikes 2.43 ms (IQR0.4-4.23 ms) and for the DN spikes was 2.43 ms (IQR 0.4-4.17 ms).

**Figure E2**. Shows the distribution of the ISI of the two spike trains together for the Ripple and FR band. For the HFO, the median ISI was 1.17 ms (IQR 0.54-6.5 ms) for the ripple band and 0.6 ms (IQR 0.3-2.6 ms) for the FR band. For the noise, the median ISI was 4.03 (IQR 0.66-10.3 ms) for the ripple band and 2.43 ms (IQR 0.4-4.2 ms) for the FR band.


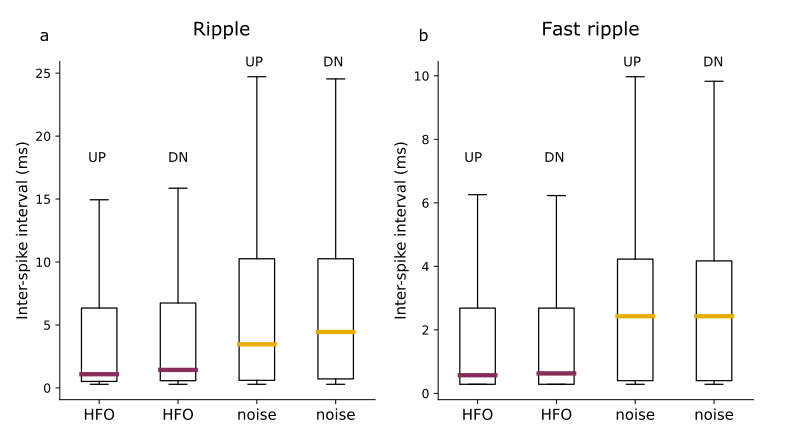


**Figure E1.** Differences in the ISI distribution of UP and DN spikes in the ripple (a) and FR band (b) between HFO and noise samples in the iEEG training signal.


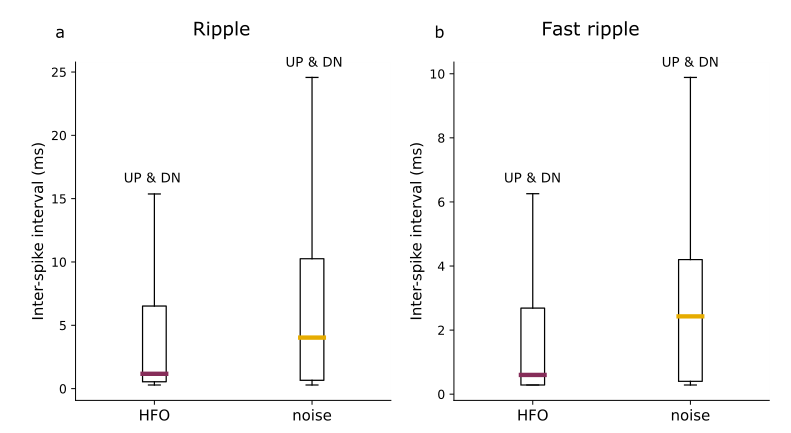


**Figure E2.** Differences in the ISI distribution the combined spike trains in the ripple (a) and FR band (b) between HFO and noise samples in the iEEG training signal.

**Setting the initial parameters of the core SNN**

The results of the ISI analysis was used to set the initial parameters of the core SNN. We constructed a layer of 256 neurons and sent the spike trains as inputs. The time constants between the spikes form the ripple band and the second layer of neurons was drawn randomly from a normal distribution with an initial range from 0.5 to 6.3 ms (IQR). The time constants between the spikes form the FR band and the second layer of neurons was drawn randomly from a normal distribution with an initial range from 0.3 to 2.7 ms (IQR).

We analyzed the activity of the neurons in the second layer using these parameters and observed that although this range of time constants resulted in neurons generating spikes in response to some HFO in the iEEG training signal, some other neurons also responded to the noise samples, and some others did not respond at all. As an example of these different type of neurons, the upper panel of **Figure E3** shows the raster plot of 25 neurons from the second layer of the core SNN for a duration of 2 seconds from the training signal. The gray shades indicate marked HFO regions. The bottom panel of **Figure E3** shows the synaptic current of a neuron that generated spikes in response to some HFO samples in the training signal. The synaptic current increased due to the excitatory UP spikes and decreased due to the inhibitory DN spikes. We observed that only with the right balance between the excitatory and inhibitory synaptic time constants, the synaptic current would surpass the spiking threshold of the neuron. Therefore, this right balance resulted in output spikes during an HFO and not during noise. Note that we do not expect a single neuron to respond to all the HFO in the training signal since we assumed its synaptic parameters are tuned to only some frequencies that match the peak frequency of some HFO.


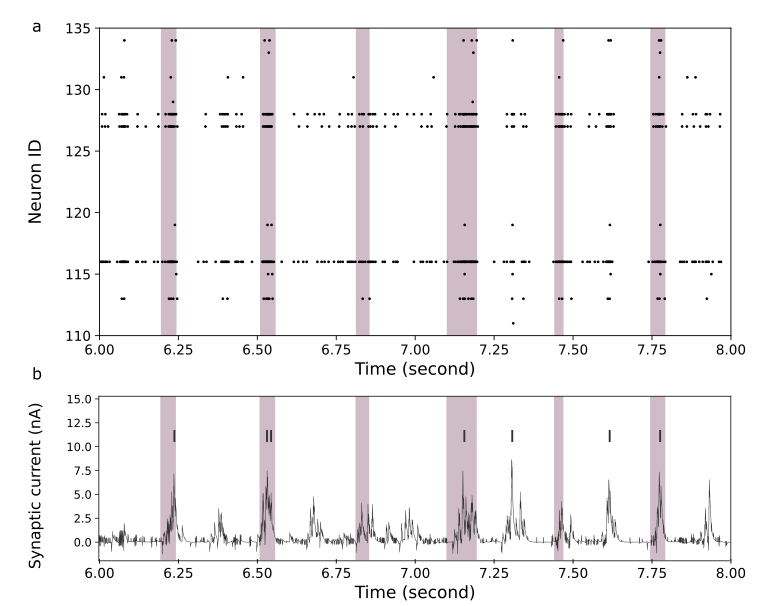


**Figure E3**. Response of second layer neurons to the iEEG training signal. (a) Raster plot showing the activity of 25 neurons of the second layer of the core SNN. The purple marks correspond to the periods of time where an HFO was marked by the Morphology detector. (b) Synaptic current of a single neuron in the second layer that respond to some HFO in the training signal and remains silent otherwise.

**Adjusting the range for the synaptic time constants**

We aim to find the time constants that minimize the number of neurons that spike during noise or that do not spike at all, and maximize the number of neurons that spike during an HFO. To asses this problem, we first classified the three type of neurons automatically. Then, we gathered the synaptic parameters of all the neurons (weight and time constant), and explored different combinations that allowed us to separate the parameters that resulted in spiking activity in the second layer during HFO and minimized spiking activity of the neurons during noise. For each neuron in the second layer, we counted the times it responded to noise and to HFO. Responding to an HFO was considered a correct response, while responding to a noise sample was considered a mistake. We set a threshold of 20% error, if a neuron responded to more than 20% of the noise samples in the training signal, it was classified as ‘noisy neuron’. If a neuron responded to some HFO samples and to less of 20% of the noise samples, it was classified as ‘HFO-sensitive neuron’. If a neuron did not produce any output spike, it was classified as ‘silent neuron’. We clustered the different type of neurons by plotting different relationships between the synaptic time constants. For example, in **Figure E4a** we plot the relationship between the UP (excitatory) and the DN (inhibitory) time constants used for the connections between the input spike trains from the signal filtered in the ripple band and the second layer neurons. Each dot in the plot represents a neuron. The *x* coordinate is the synaptic time constant between the neuron and the input UP spikes and the *y* coordinate is the synaptic time constant between the neuron and the input DN spikes. The different colors and sizes of the dots represent the three type of neurons. The smallest and light pink ones are the silent neurons, the middle size and orange are the noisy neurons, and the biggest and darker pink ones represent the HFO-sensitive neurons. We observed that there is no clear way to separate the neurons with respect to the excitatory and inhibitory time constants from the input signal filtered in the ripple band. However, for the inputs coming from the signal filtered in the FR band (**Figure E4b**), we observed that a time constant greater than 6 ms resulted in more HFO-sensitive neurons.


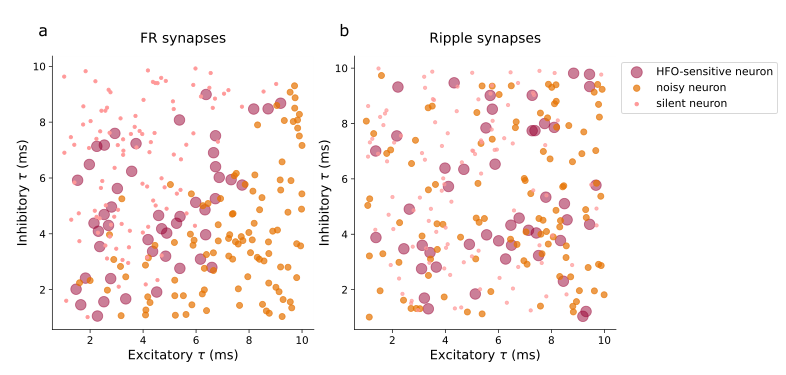


**Figure E4**. Relation between the excitatory and inhibitory time constant for each neuron in the second layer of the core SNN. While in the ripple band we cannot see a clear difference between the HFO-sensitive, noisy and silent neurons (a), in the FR band we can identify the parameters that result in more HFO-sensitive neurons (b).

We then analyzed other interactions between the excitatory and the inhibitory time constants. For example, **Figure E5** shows that either by subtracting or dividing the excitatory and inhibitory time constants, we can differentiate the three type of neurons for both inputs, the signal filtered in the ripple and FR band. To maximize the HFO-sensitive neurons, the relationship between the excitatory and the inhibitory time constant should be kept within a range. A larger time constant from the excitatory inputs with respect to the inhibitory ones results in more noisy neurons, while a larger time constant from the inhibitory inputs time constants with respect to the excitatory ones results in more silent neurons.

Based on these observations we adjusted the parameters of the SNN. We set a range for the excitatory time constant to 3-6 ms. For each neuron in the perceptron layer, we draw a random value within this range to set the excitatory time constant. To keep the relationship between the excitation and inhibition, the inhibitory time constant of each neuron depended on the excitatory one. For each neuron in the perceptron layer, the inhibitory time constant was set to 0.1 to 1 ms smaller than its excitatory time constant. We repeated the cluster analysis with the new parameters and observed that we could no longer establish a clear separation. Nonetheless, the last set of parameters lead to the detection of enough clinically relevant HFO.


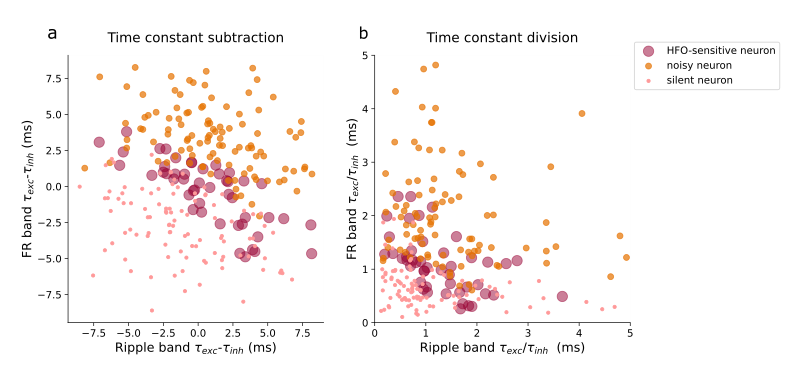


**Figure E5**. Relation between the combination of the ripple and FR synapses. Either by subtracting the excitatory time constant from the inhibitory one (a) or by dividing the excitatory time constant by the inhibitory one (b), we can identify the parameter relationships that result in more HFO-sensitive neurons.

**Values for the synaptic weights of the core SNN**

We evaluated the effect of the synaptic weight. We first chose a weight that generated some activity in the SNN without overexciting the neurons. This means that the weight should not be strong enough to cause a neuron to spike after a single input spike but rather it should need few input spikes before generating output spikes. We explored different values for the weights (1, 2, and 3 nA). The value of the weight did not have as much influence on detecting HFO as the time constants. However, we observed that for each neuron, the synaptic weight it receives from the UP spikes should have the same strength as the one it receives from the DN spikes.

**Setting the synaptic parameters of the in-band artifact rejection SNN**

To set the synaptic parameters of the global inhibitory neuron and the dis-inhibitory neuron from the artifact rejection SNN, we filtered the ECoG training signal in the FR band and converted it to UP and DN spikes. We then analyzed the differences between the signal containing an HFO and the signal containing fast transients by comparing the temporal characteristics (i.e. ISI) of the UP and DN spike trains obtained when the signal was converted into spikes. We defined a burst of UP spikes followed by a burst of DN spikes as ‘UP-DN cycle’. **Figure E6b** shows how the median amount of UP-DN cycles of a fast transient was smaller (2 cycles) than the median amount of cycles during an HFO (6 cycles). We could also observe that the median duration of an artifact was shorter (8 ms) (**Figure E6c**) than the median HFO duration (24 ms) (**Figure E6a**). Nevertheless, the median duration of a single cycle during a fast transientlasts longer (3.2ms) than a single cycle during an HFO (2.6ms). The cycle characteristics were used to select the parameters of the dis-inhibitory and global-inhibitory neurons. Therefore, to suppress the activity of the second layer neurons in the first UP-DN cycle of the input signal, we set a time constant of 5 ms for the synapse between the global-inhibitory neuron and the second layer neurons.


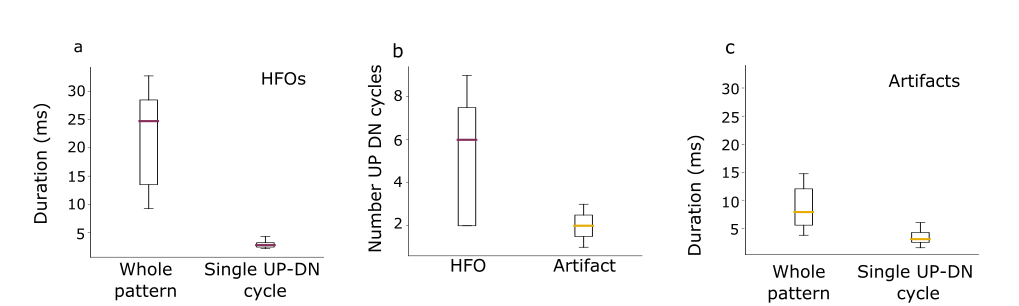


**Figure E6**. (a) The inputs spike train during an HFO lasted 24 ms (median), with a single cycle lasting 2.6 ms (median). (b) The HFO comprise more UP-DN cycles (median 6 cycles) than the artifacts (median 2 cycles). (c) The input spike trains during an artifact lasted 9 ms (median), with a single cycle lasting 3.2 ms (median).

The same time constant (5 ms) was used for the connections between the input spike trains and the dis-inhibitory neuron. This decision was taken so that the dis-inhibitory neuron could react fast and start the dis-inhibition stage when an HFO is present in the signal. Note that once the dis-inhibitory neuron suppresses the global inhibitory neuron, it should keep this inhibition during the average duration of a single HFO. We chose a time constant of 20 ms for the connections between the dis-inhibitory and global-inhibitory neurons.
